# The association of statin use with the risk of anxiety: a systematic review and meta-analysis

**DOI:** 10.3389/fpsyt.2026.1769044

**Published:** 2026-02-18

**Authors:** Yutong Yan, Sijing Deng, Ying Li, Jie Fu, Hongxia Gong, Haiyan Zhang, Feng Zhang

**Affiliations:** 1 School of Health and Wellbeing, University of Glasgow, Glasgow, United Kingdom; 2 Department of Cardiology, Daping Hospital, Army Medical University, Chongqing, China

**Keywords:** anxiety, anxiety disorders, meta-analysis, statins, systematic review

## Abstract

**Background:**

Statins are widely prescribed for the primary and secondary prevention of cardiovascular diseases, given their efficacy in lowering low-density lipoprotein cholesterol levels and reducing the risk of cardiovascular events. However, statins may exert effects on the central nervous system. Previous studies have hypothesized that statin use may exert protective effects against anxiety via mechanisms including anti-inflammatory activity and improved endothelial function. Conversely, observational studies have also reported associations between statin use and increased anxiety symptoms. To date, findings on the association between statin use and anxiety risk have been inconsistent. Therefore, this systematic review and meta-analysis aimed to systematically search and assess the available evidence, clarify the association between statin use and the risk of anxiety, and offer evidence-based guidance for clinical practice.

**Methods:**

We searched PubMed, Web of Science, Embase, and the Cochrane Library for studies investigating the association between statin use and anxiety risk, with the search period ranging from the inception of each database to January 2026. Studies were screened against predefined inclusion criteria, and relevant data were extracted. The Risk of Bias in Non-randomized Studies of Interventions (ROBINS-I) tool was employed to assess the risk of bias in included non-randomized studies. Hazard ratios (HRs) and 95% confidence intervals (CIs) were pooled with a random-effects model.

**Results:**

A total of five studies with 1,919,059 participants were included. The meta-analysis showed that statin use was not associated with an increased risk of anxiety disorders (HR = 0.75,95% CI (0.55,1.04), *P = 0.08)*. Subgroup analyses revealed that statin use was associated with a reduced risk of anxiety only in studies with a sample size < 10,000 (*P < 0.05*). No significant association was observed in other subgroups, including those stratified by region or study design, notably, studies with a sample size > 10,000 also showed no significant effect (P > 0.05).

**Conclusion:**

The existing evidence suggests that statin use may not be associated with the risk of developing anxiety. Given the limitations of this study, future large-scale, multi-center, and prospective cohort studies are warranted to validate these findings.

**Systematic review registration:**

https://www.crd.york.ac.uk/prospero/, identifier PROSPERO CRD420261291901.

## Introduction

Anxiety disorders represent a prevalent category of mental illnesses (1), characterized by excessive worry, fear and tension that severely impair patients’ daily functioning and social interactions (2). Large-scale epidemiological studies have shown that anxiety disorders severely impair patients’ daily functioning and social interactions at some point in their lives (3). Currently, approximately 301 million people worldwide suffer from anxiety disorders, making it the second most common mental disorder after depression (4). Compared with the general population, individuals with anxiety disorders face a substantially higher risk of mortality from both natural and unnatural causes (5). Consequently, anxiety disorders have imposed an economic burden and social pressure on numerous countries and regions (6–8). Despite the diverse clinical manifestations of anxiety disorders, their diagnosis and treatment are hindered by an incomplete understanding of their etiologies and pathological mechanisms.

Statins are among the most widely prescribed medications globally, utilized for the primary and secondary prevention of cardiovascular events (9). However, their potential neuropsychiatric side effects, particularly the association with anxiety risk, remain controversial (10, 11). On the one hand, statins are hypothesized to exert neuroprotective effects and may reduce anxiety risk via mechanisms such as anti-inflammatory activity, antioxidative effects, and modulation of neurotransmitter systems (12, 13). On the other hand, observational studies have suggested that statin use may be associated with exacerbated anxiety symptoms, possibly mediated by interference with cholesterol metabolism and neurotransmitter systems (14–17).

Although previous meta-analyses have explored the association between statins and anxiety, suggesting a potential anxiety-reducing effect (18), recent large-scale cohort studies and updated analyses have reported no significant associations (19–21). This inconsistency not only exists among observational studies but also extends to higher-level evidence syntheses, leading to ambiguity in clinical decision-making. Previous meta-analyses examining the statin-anxiety association included only two studies, which may have had limited statistical power. Methodologically, these studies failed to adopt the currently recommended ROBINS-I tool for rigorous bias assessment of observational studies. To address these gaps in evidence and methodology, the present study conducted an updated systematic review and meta-analysis incorporating the latest literature. This study aimed to comprehensively search and evaluate existing evidence, clarify the association between statin use and anxiety risk, and provide evidence-based guidance for clinical medication decisions.

## Methods

This meta-analysis was conducted in accordance with the Preferred Reporting Items for Systematic Reviews and Meta-Analyses guidelines (22–24). The study protocol was retrospectively registered in PROSPERO (Registration number: CRD420261291901).

### Search strategy

A systematic literature search was performed in PubMed, Web of Science, Embase, and the Cochrane Library. The search period covered all publications from the establishment of each database up to January 2026, with no language restrictions. Additionally, reference lists of included studies and relevant systematic reviews were manually searched to identify additional eligible publications. Search terms related to statins and anxiety risk included: statin, statins, atorvastatin, simvastatin, anxiety, anxiety disorders, risk, incidence, association, etc. Taking PubMed as an example, we elaborately listed the search terms for the association between statins and the risk of anxiety, and performed the retrieval with Boolean logic operators. The detailed search strategy is provided in the supplementary material (**Supplementary Table S1**).

### Inclusion and exclusion criteria

Inclusion criteria:

(1) Observational study design (cross-sectional, case-control, or cohort study);
(2) Comparison between participants receiving statin therapy (experimental group) and those not receiving statin therapy (control group);
(3) Anxiety defined using standardized questionnaires (e.g., Generalized Anxiety Disorder-7 Scale [GAD-7], Hospital Anxiety and Depression Scale-Anxiety Subscale [HADS-A], State-Trait Anxiety Inventory [STAI]) or medical record-derived diagnostic codes/anti-anxiety medication prescriptions (e.g., selective serotonin reuptake inhibitors, benzodiazepines);
(4) Provision of effect sizes (e.g., HR, odds ratio) with 95% CIs.

Exclusion criteria:

(1) Insufficient or invalid data provided;
(2) Case report, comments, reviews, systematic review.
(3) Duplicate publications.

### Data extraction

Two independent reviewers screened the studies, extracted data, and cross-validated the results. Disagreements were resolved through discussion and consensus. Extracted data included: author names and publication year, country of origin, study design, sample size, age, gender distribution, anxiety assessment tools, follow-up duration, adjustment factors, and effect sizes.

### Risk of bias assessment

The ROBINS-I tool was used to assess the risk of bias in included non-randomized intervention studies. Each study was evaluated across seven domains: confounding, selection of participants, classification of interventions, deviations from intended interventions, missing data, measurement of outcomes, and selection of reported results. The overall risk of bias for each study was determined based on the highest bias level identified across these domains and classified as low, moderate, or high according to established criteria. Two independent researchers conducted the assessments, with disagreements resolved through discussion.

### Statistical analysis

Statistical analysis was performed using RevMan 5.3. HRs and its 95% CI were used as effect measures to evaluate the association between the statin use and the anxiety risk. Heterogeneity among the studies was assessed using the χ2 test and I*^2^*. If *P > 0.10 and I^2^ < 50%*, heterogeneity was considered acceptable, and a fixed-effects model was used for analysis. If *P ≤ 0.10 and I^2^ ≥ 50%*, significant heterogeneity was assumed, and a random-effects model was employed. Subgroup analyses were conducted based on region (Asian vs Non-Asian), study design (Retrospective vs Prospective), and sample size (<10,000 vs >10,000). Sensitivity analyses were performed by changing the effect model and excluding studies with high risk of bias to assess the stability of the results. Funnel plot analysis for publication bias was planned if ≥ 10 studies were included.

## Results

### Literature search

**Figure 1:**
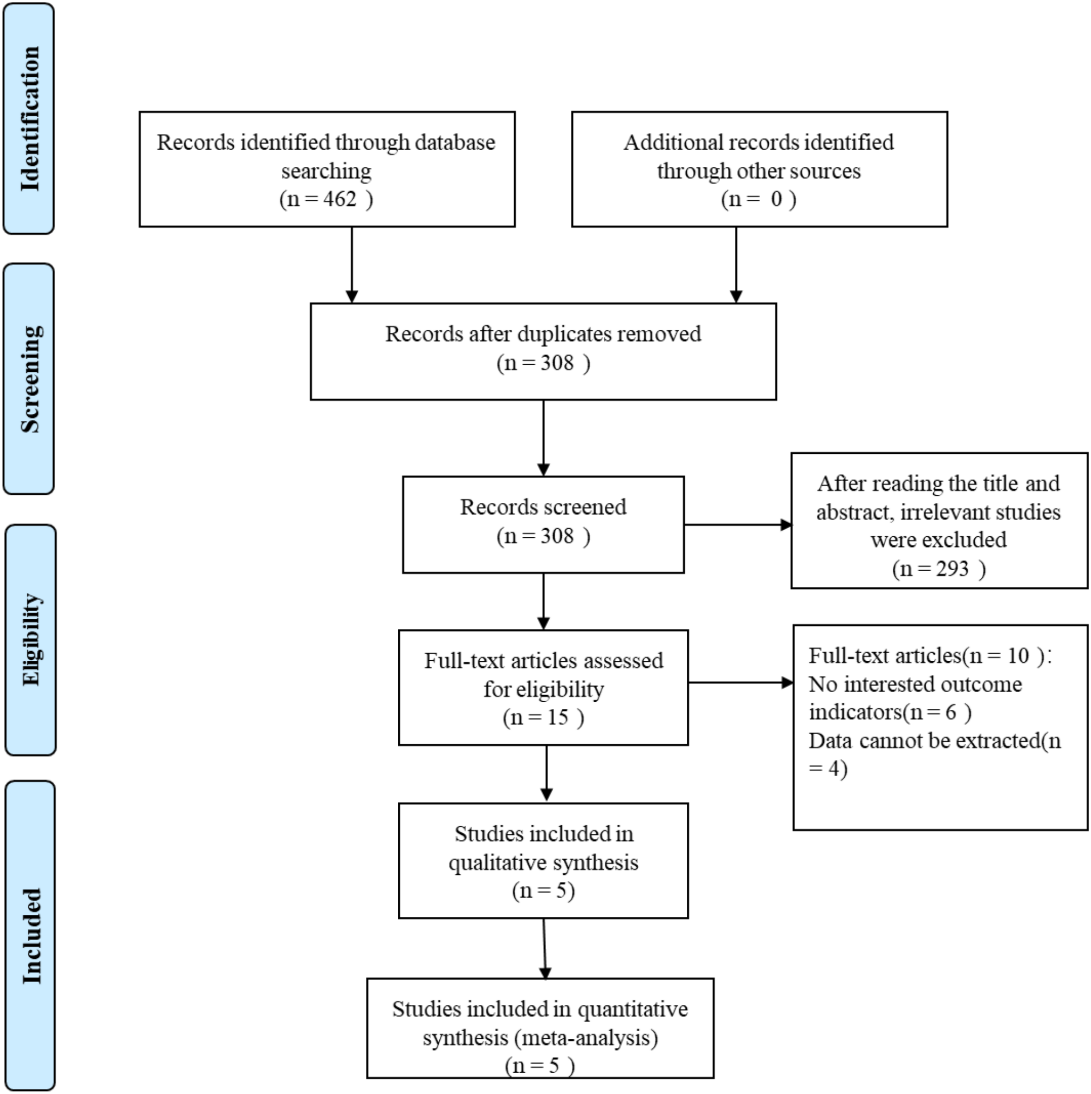
Flow chart of article selection process.

The initial database search yielded 462 articles. After rigorous screening, five literatures were finally included for a meta-analysis (**Figure 1**).

### Study characteristics

The five included studies involved a total of 1,919,059 participants. Two studies adopted a prospective design, and three were retrospective design. Two studies were conducted in China, while the remaining ones were carried out in the UK, the US, and Sweden. The sample sizes of these studies ranged from 371 to 1,149,384. Detailed characteristics of the included studies are summarized in **Table 1**. Risk of bias assessment using the ROBINS-I tool revealed that four studies had a moderate overall risk of bias, and one study had a high risk of bias (**Table 2**).

**Table 1:** Characteristics of included studies.

References	Study design	Data source	Country	Sample size	Age(median/mean)	Male sex (%)	Anxiety outcome	Follow-up period	Adjustment factors
Molero et al. (19)	Retrospective cohort study	Swedish nationwide registers	Swedish	1,149,384	Not report	625,616 (54.4%)	F40–F45, F48	8 Years	1. Demographic statistics and socio-economic factors: Age
Yang et al. (20)	Prospective cohort study	UK Biobank	UK	363,551	56.73	173,016(47.6%)	F40-F41,Generalized Anxiety Disorder	13 Years	1. Demographic statistics and socio-economic factors: Age, Sex, Education, Ethnicity, Townsend Deprivation Index, Average Household Income. 2. Lifestyle and behavioral factors: Body Mass Index, Smoking Status, Drinking Status, Physical Activity. 3. Dietary factors: Fruit Intake, Vegetable Intake, Fish Intake, Red Meat Intake, Processed Meat Intake, Whole Grains, Dairy Products. 4. Clinical diseases and historical factors: Diabetes, High Blood Pressure, Heart Problem, Cerebrovascular Disease, Dyslipidemia, Cancer, Chronic Lung Disease, Other Psychiatric Disorders, Family History of Depression. 5. Drug combination factors: Antihistamines, Beta Blockers, Nonsteroidal Anti-inflammatory Drugs, Acetaminophen, Opioids, Systemic Glucocorticoids, Psychotropic Medications.
Ye et al. (21)	Case-control study	Hong Kong Clinical Data Analysis & Reporting System	China	396,614	58.43	205,625 (51.85%)	ICD-9-CM	10 Years	Not report
Yeh et al. (25)	Retrospective cohort study	Taiwan’s National Health Insurance Research Database	China	9,139	65.1	5,630(61.60%)	ICD-9-CM	The mean follow-up durations were 7.74 and 5.09 years for the statin and nonstatin users	1. Demographic statistics and socio-economic factors: Age, Sex. 2. Clinical diseases and historical factors: Sleep Disorders, Diabetes, Hypertension, Hyperlipidemia, Alcohol-related Illnesses, Chronic Kidney Disease, Coronary Artery Disease, Stroke, Cancer. 3. Drug combination factors: Inhaled Corticosteroids, Oral Steroids.
Young-Xu et al. (26)	Prospective cohort study	Lown Cardiovascular Center	America	371	Not report	Not report	Kellner Symptom Questionnaire	7 Years	1. Demographic statistics and socio-economic factors: Age, Gender, Education. 2. Study Design and Follow-up Factors: Length of Follow-up, Statin Usage Prior to Study Entry. 3. Physiological and biochemical indicators: Blood Glucose Level, Systolic Blood Pressure, Diastolic Blood Pressure, Total Cholesterol, High-density Lipoprotein, Heart Rate. 4. Lifestyle and behavioral factors: Current Smoking, Regular Exercise, Alcohol Use. 5. Psychosocial factors: Past Major Life Events, Anticipated Major Future Life Events. 6. Drug combination factors: Use of Antidepressants at Time of Enrollment, Use of Anti-anxiety Drugs at Time of Enrollment, Use of Beta-blockers at Enrollment and During Follow-up, Use of Calcium Channel Blockers at Enrollment and During Follow-up. 7. Clinical diseases and historical factors: History of Catheterization, History of Myocardial Infarction, History of Hypertension, History of Diabetes, Incidence of Myocardial Infarction During Follow-up, Incidence of Stroke During Follow-up, Incidence of Catheterization During Follow-up, Incidence of Revascularization During Follow-up.

**Table 2:** Summary of bias assessment using the ROBINS-I tool.

References	D1	D2	D3	D4	D5	D6	D7	Overall bias
Molero et al. (19)	Low	Low	Low	Low	Low	Moderate	Low	Moderate
Yang et al. (20)	Low	Low	Low	Low	Low	Low	Moderate	Moderate
Ye et al. (21)	High	Low	Low	Low	Low	Low	Low	High
Yeh et al. (25)	Low	Moderate	Low	Low	Moderate	Moderate	Low	Moderate
Young-Xu et al. (26)	Moderate	Low	Low	Low	Low	Low	Low	Moderate

Domains:

D1: Bias due to confounding.

D2: Bias in selection of participants.

D3: Bias in classification of exposures.

D4: Bias due to deviations from intended exposures.

D5: Bias due to missing data.

D6: Bias in measurement of outcomes.

D7: Bias in selection of the reported result.

Red indicates high risk, green indicates low risk, and light yellow indicates moderate risk.

### Main analysis

**Figure 2:**
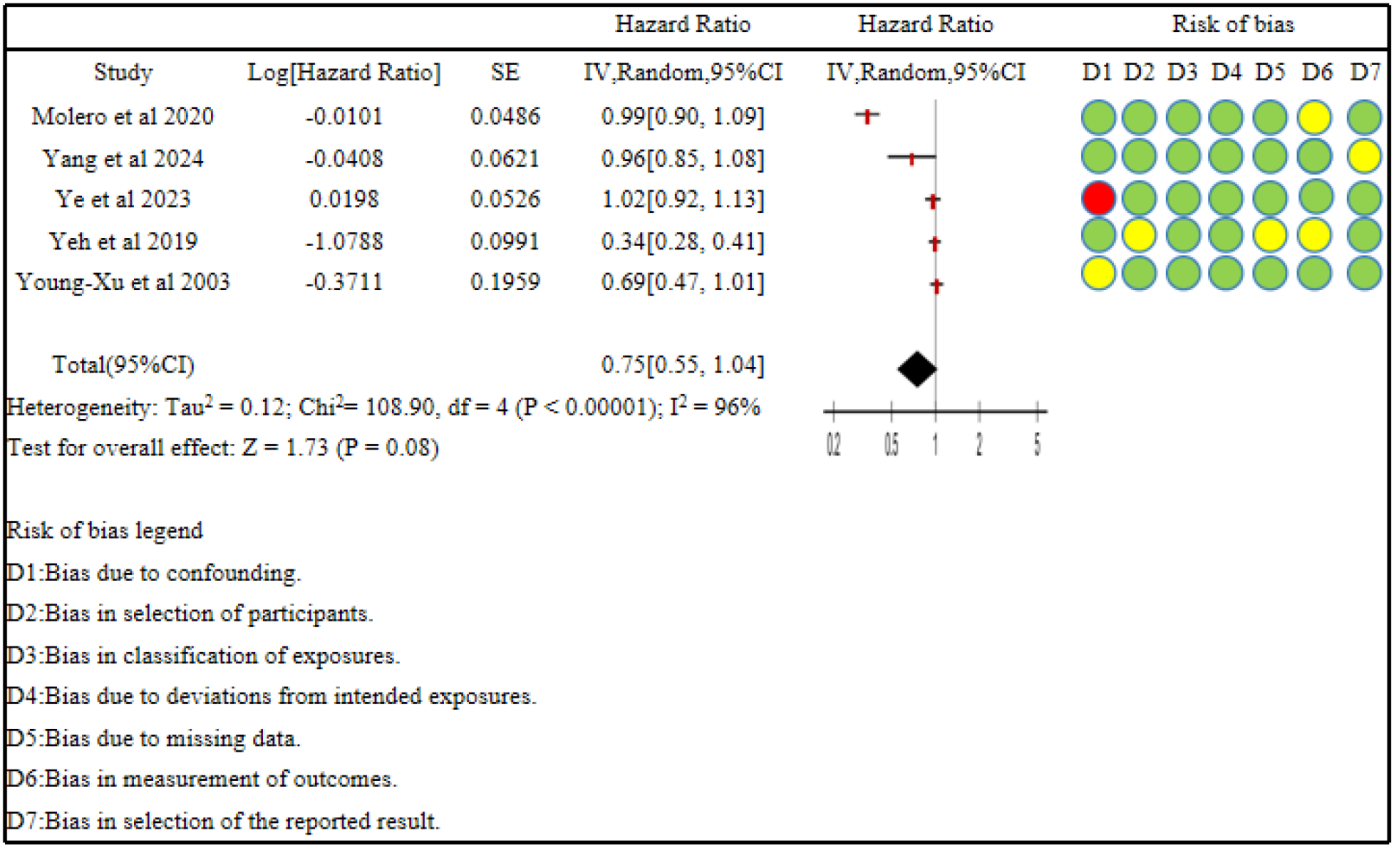
Forest plot of the association between statin use and anxiety risk. Red indicates high risk, green indicates low risk, and light yellow indicates moderate risk.

Heterogeneity testing indicated significant heterogeneity among the included studies (*P < 0.00001, I^2^ = 96%*), so a random-effects model was used for pooling. The meta-analysis showed that statin use was not associated with an increased risk of anxiety disorders (HR = 0.75, 95% CI (0.55,1.04), *P = 0.08*) (**Figure 2**).

### Subgroup analysis

Subgroup analysis based on region (Asian vs. Non-Asian), study design (retrospective vs. prospective), and sample size (<10,000 vs >10,000) are presented in **Table 3**. Statin use was associated with a reduced anxiety risk only in studies with a sample size < 10,000 (*P < 0.05*). No significant associations were observed in subgroups stratified by region (Asian vs. Non-Asian), study design (retrospective vs. prospective), or sample size > 10,000 (*P > 0.05*).

**Table 3:** Subgroup analysis.

Subgroup analysis	No. of studies	Heterogeneity		HR(95%CI)	P value
		I^2^	P		
Study design					
Retrospective	3	98.00%	< 0.00001	0.71(0.43,1.15)	0.165
Prospective	2	63.20%	< 0.00001	0.85(0.63,1.16)	0.319
Sample size					
<10000	2	90.70%	0.001	**0.48(0.24,0.95)**	**0.036**
>10000	3	0.00%	0.760	0.99(0.94,1.05)	0.817
Region					
Asian	2	98.90%	< 0.00001	0.59(0.20,1.73)	0.338
Non-Asian	3	41.60%	0.181	0.97(0.90,1.04)	0.356

The bold font indicates statistically significant differences.

### Sensitivity analysis

**Figure 3:**
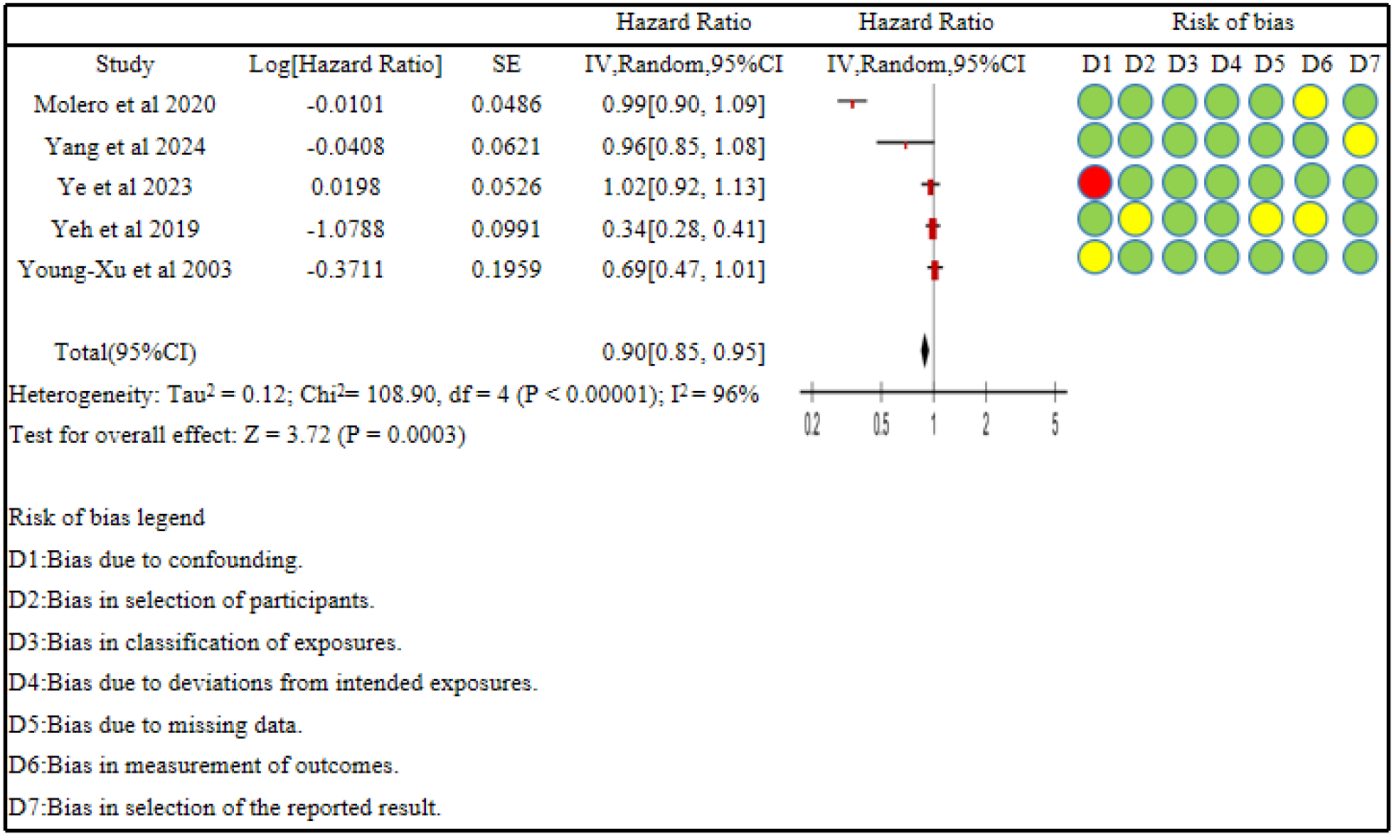
Forest plot for sensitivity analysis using the fixed-effects model. Red indicates high risk, green indicates low risk, and light yellow indicates moderate risk.

Sensitivity analyses by changing the effect model showed that under the fixed-effects model, statin use was associated with a reduced risk of anxiety (**Figure 3**). After excluding the study by Ye et al., the results indicated no significant association between statin use and anxiety risk (HR = 0.69,95% CI (0.44,1.08), *P = 0.11*), suggesting instability of the meta-analysis results.

### Publication Bias

Given the small number of included studies (n = 5), funnel plot analysis for publication bias was not feasible.

## Discussion

This systematic review and meta-analysis synthesized data from five studies involving nearly 2 million participants, revealing no significant association between statin use and anxiety risk. This finding contradicts the hypothesis that “statins may reduce anxiety” proposed by some previous observational studies (25, 26) but is consistent with the results of most high-quality cohort studies, including a Swedish total-population cohort study that also reported no significant association between statin use and anxiety risk (19, 20). Furthermore, a recent meta-analysis on depression and inflammation also indicated that statin use for improving depressive symptoms did not increase anxiety risk (14).

In the present study, the pooled HR point estimate leaned toward a protective effect (HR < 1) but did not reach statistical significance, indicating that existing evidence is insufficient to confirm whether statins exert significant protective or harmful effects on anxiety. The meta-analysis showed substantial heterogeneity, and sensitivity analyses revealed that statin use might reduce the risk of anxiety under the fixed-effects model. This uncertainty may arise from heterogeneity in population characteristics, statin types and doses, anxiety assessment methods, and follow-up durations across studies. Evidence on the impact of statin use on the pathogenesis of mood disorders remains inconsistent: some studies report associations between statin use and mood disorders, while others suggest beneficial effects on mental health (14). A systematic meta-analysis of randomized, double-blind, placebo-controlled trials indicated that adjunctive statin therapy may be beneficial for treating depressive symptoms (27). Another study showed that statin use could reduce the risk of depression and anxiety in patients with coronary artery disease (26). Statins are potent cholesterol-lowering agents that also inhibit endotoxin-induced inflammatory responses and reduce lipopolysaccharide-induced monocyte cytokine production (28). Therefore, their anti-inflammatory and antioxidant properties may contribute to the treatment of depression and improving mood (29). A systematic review and meta-analysis on the impact of statins on mental health provided partial support for the emotional benefits of these drugs (18). A retrospective cohort study investigated the effect of statins on anxiety and major depression in patients with asthma-chronic obstructive pulmonary disease overlap syndrome found that statin use significantly reduced the risk of anxiety and major depression (25).

Subgroup analyses revealed a statistically significant reduction in the risk of anxiety only in small-sample studies (< 10,000 participants), which may explain the conflicting results in the previous literature. Notably, the two small-sample studies included in this meta-analysis were rated as having a moderate risk of bias, while among the three large-sample studies, one had a high risk of bias. This suggests that studies reporting “protective effects” may be more susceptible to methodological limitations. Small-sample studies may have greater variability or limitations in population selection, control of confounding factors (e.g., severity of cardiovascular disease, comorbid psychiatric history), or outcome assessment. These biases may systematically underestimate risks or overestimate protective effects. The smallest study (26) (26), despite extensive adjustments, was limited to a specific population from a single cardiovascular center, restricting generalizability. In contrast, the largest study with a relatively low risk of bias (19) (19) provided an estimate closer to a null effect, which may better reflect the true association in the general population after adequate confounding control. Therefore, inconsistencies in previous observational studies may partly be attributed to differences in study size and methodological quality. This emphasizes the need for caution when interpreting observational evidence in this field and prioritizing data from large-scale, high-quality studies. Furthermore, variations in anxiety assessment methods across studies may have influenced the results: some studies (19, 21, 25) relied on medical diagnostic codes (more likely to identify clinically significant anxiety disorders), while others (20, 26)used standardized anxiety symptom scales (more sensitive to subclinical or mild symptoms). Unfortunately, due to the limited number of eligible studies, subgroup analysis based on assessment methods was not feasible to examine this potential impact. Future studies should detail anxiety assessment tools and definitions to facilitate more refined comparisons and syntheses.

Animal studies have provided insights into the potential anti-anxiety mechanisms of statins. A study evaluating the effects of different statins in a Wistar rat anxiety model showed that atorvastatin, rosuvastatin, and simvastatin all exerted significant anti-anxiety effects compared with the control group (30). Santos et al. (31) reported that treatment with simvastatin and/or fluoxetine alleviated anxiety-like behaviors in adult rats in the elevated plus maze and open field tests. Statins may alleviate anxiety symptoms through multiple mechanisms: inhibiting pro-inflammatory factors, regulating serotonin and neurotransmitter metabolism, improving cerebral blood flow and endothelial function, and upregulating neuroprotective factors. Inflammation is recognized as a key pathophysiological basis for mood disorders such as anxiety and depression (32). Anxiety is often accompanied by activation of the inflammatory response, and elevated levels of inflammatory factors (e.g., tumor necrosis factor-α, interleukin-6) may affect neurotransmitter metabolism and neuroplasticity, thereby exacerbating anxiety (32). Statins may reduce chronic inflammation-induced or -exacerbated anxiety by inhibiting multiple pro-inflammatory cytokines (33). Cholesterol is an important precursor for neurotransmitter synthesis (e.g., serotonin, dopamine). While statins lower cholesterol levels, they may also modulate neurotransmitter synthesis and metabolism.

Studies have shown that statins may increase cerebral serotonin concentrations by regulating serotonin transporter function, exerting anti-anxiety effects (34). Additionally, statins may upregulate N-methyl-D-aspartate receptors to improve emotional behavior (13). Statins can improve vascular endothelial function, increase cerebral blood perfusion, and provide sufficient oxygen and nutrients to the brain, supporting normal neural cell function (35). Furthermore, statins may promote neural cell repair and regeneration by regulating the expression of neuroprotective factors such as brain-derived neurotrophic factor, exerting positive effects on anxiety-related neural circuit function (36).

Despite these potential mechanisms, the link between biological plausibility and clinical observations must be interpreted with caution. Existing evidence does not indicate that statins increase anxiety risk, so statin therapy should not be avoided or delayed due to concerns about inducing anxiety. The established benefits of statins in the primary and secondary prevention of cardiovascular diseases should be the primary consideration in clinical decision-making. For patients with anxiety disorders or anxiety proneness who require statin therapy, clinicians should inform them of the current evidence to alleviate unnecessary concerns. Routine monitoring of emotional status during treatment is a reasonable clinical practice. If patients report new or exacerbated anxiety symptoms, a comprehensive assessment should be conducted to consider multiple potential causes (e.g., underlying diseases, other medications, life stressors) rather than presuming statins as the cause.

### Limitations

This study has several limitations. First, limited data on specific statin types (e.g., atorvastatin, simvastatin), doses, treatment durations, and medication adherence precluded in-depth analyses of these factors, limiting the precision of the conclusions. Differences in the pharmacokinetic properties of statins (e.g., lipophilicity, blood-brain barrier permeability, anti-inflammatory potency) may affect cholesterol metabolism, inflammatory responses, and neurotransmitter function in the central nervous system, resulting in varying effects on anxiety risk. Additionally, differences in doses and treatment durations may result in varying cumulative or threshold effects, which are potential sources of heterogeneity in observational studies and the present meta-analysis. Future studies should conduct well-designed, large-scale prospective cohort studies and systematically collect and report detailed statin treatment information. Secondly, although most studies adjusted for key confounding factors, inconsistencies in covariate adjustment across studies cannot fully exclude residual confounding. Thirdly, the meta-analysis exhibited high heterogeneity. Although subgroup and sensitivity analyses were conducted to explore and control for heterogeneity, the complex and diverse sources of heterogeneity (e.g., differences in baseline cardiovascular disease comorbidity across study populations) rendered complete elimination difficult. A neglected core potential source lies in discrepancies in the baseline health status of the study populations, particularly with respect to the comorbidity profile of cardiovascular disease (CVD). Statins are firstly indicated for the primary and secondary prevention of CVD, and thus individuals receiving statin therapy inherently have a higher risk of CVD or a confirmed diagnosis of CVD. Notably, CVD and its severity are significantly associated with an elevated risk of anxiety and depression. The included studies in the present meta-analysis exhibited inconsistencies in the definition and adjustment of CVD status, which may have contributed to the heterogeneity in effect sizes across individual studies and also represents a key driver of the high heterogeneity observed in this meta-analysis, further limiting the certainty of our conclusions. Finally, although the primary analysis using a random-effects model yielded a non-significant association between statin use and anxiety risk, the pooled estimate shifted to a statistically significant reduction in risk when a fixed-effects model was applied instead. The random-effects model is generally regarded as a more conservative approach for addressing heterogeneity. Given the small number of included studies and the aforementioned limitations, the null finding of no significant association in the present study must be interpreted with extreme caution.

## Conclusion

Current evidence indicates that statin use is not associated with an increased risk of anxiety disorders. Future large-scale, prospective, multi-center cohort studies are warranted to further validate this finding.

## Data Availability

The original contributions presented in the study are included in the article/**Supplementary material**, further inquiries can be directed to the corresponding author/s.

## References

[1] Lakhawat SS Mech P Kumar A Malik N Kumar V Sharma V . Intricate mechanism of anxiety disorder, recognizing the potential role of gut microbiota and therapeutic interventions. Metab Brain Dis. (2024) 40:64. doi: 10.1007/s11011-024-01453-1, PMID: 39671133

[2] Zhang B Li Y Chu W Li Y Zhang J Lv Z . Efficacy of non-pharmacological interventions for alleviating insomnia in individuals with generalized anxiety disorder: systematic evaluation and net meta-analysis. Front Psychiatry. (2025) 16:1669888. doi: 10.3389/fpsyt.2025.1669888, PMID: 41244878 PMC12615457

[3] Bandelow B Michaelis S . Epidemiology of anxiety disorders in the 21st century. Dialogues Clin Neurosci. (2015) 17:327–35. doi: 10.31887/DCNS.2015.17.3/bbandelow, PMID: 26487813 PMC4610617

[4] GBD 2019 Mental Disorders Collaborators . Global, regional, and national burden of 12 mental disorders in 204 countries and territories 1990-2019: A systematic analysis for the global burden of disease study 2019. Lancet Psychiatry. (2022) 9:137–50. doi: 10.1016/s2215-0366(21)00395-3, PMID: 35026139 PMC8776563

[5] Meier SM Mattheisen M Mors O Mortensen PB Laursen TM Penninx BW . Increased mortality among people with anxiety disorders: total population study. Br J psychiatry: J Ment Sci. (2016) 209:216–21. doi: 10.1192/bjp.bp.115.171975, PMID: 27388572 PMC5082973

[6] Chisholm D Sweeny K Sheehan P Rasmussen B Smit F Cuijpers P . Scaling-up treatment of depression and anxiety: A global return on investment analysis. Lancet Psychiatry. (2016) 3:415–24. doi: 10.1016/s2215-0366(16)30024-4, PMID: 27083119

[7] Chodavadia P Teo I Poremski D Fung DSS Finkelstein EA . Prevalence and economic burden of depression and anxiety symptoms among Singaporean adults: results from a 2022 web panel. BMC Psychiatry. (2023) 23:104. doi: 10.1186/s12888-023-04581-7, PMID: 36782116 PMC9925363

[8] Davis LL Schein J Cloutier M Gagnon-Sanschagrin P Maitland J Urganus A . The economic burden of posttraumatic stress disorder in the United States from a societal perspective. J Clin Psychiatry. (2022) 83:1–9. doi: 10.4088/JCP.21m14116, PMID: 35485933

[9] Ghani Khan K Kaur P Bhagat M Klair HK Bakhtawar M Aldea Saldaña JM . Effect of statin therapy on clinical outcomes in patients with cardiovascular risks: A systematic review and meta-analysis. Cureus. (2025) 17:e88238. doi: 10.7759/cureus.88238, PMID: 40831859 PMC12359277

[10] Xiao X Deng H Li P Sun J Tian J . Statin for mood and inflammation among adult patients with major depressive disorder: an updated meta-analysis. Front Psychiatry. (2023) 14:1203444. doi: 10.3389/fpsyt.2023.1203444, PMID: 38034928 PMC10684957

[11] Cham S Koslik HJ Golomb BA . Mood, personality, and behavior changes during treatment with statins: A case series. Drug Saf - Case Rep. (2016) 3:1. doi: 10.1007/s40800-015-0024-2, PMID: 27747681 PMC5005588

[12] Chen J Zhang C Jiang H Li Y Zhang L Robin A . Atorvastatin induction of vegf and bdnf promotes brain plasticity after stroke in mice. J Cereb Blood Flow metabolism: Off J Int Soc Cereb Blood Flow Metab. (2005) 25:281–90. doi: 10.1038/sj.jcbfm.9600034, PMID: 15678129 PMC2804085

[13] Parent MA Hottman DA Cheng S Zhang W McMahon LL Yuan LL . Simvastatin treatment enhances nmdar-mediated synaptic transmission by upregulating the surface distribution of the glun2b subunit. Cell Mol Neurobiol. (2014) 34:693–705. doi: 10.1007/s10571-014-0051-z, PMID: 24687455 PMC4142643

[14] Adzic M Brkic Z Mitic M Francija E Jovicic MJ Radulovic J . Therapeutic strategies for treatment of inflammation-related depression. Curr neuropharmacology. (2018) 16:176–209. doi: 10.2174/1570159x15666170828163048, PMID: 28847294 PMC5883379

[15] Pop G Farcaș A Butucă A Morgovan C Arseniu AM Pumnea M . Post-marketing surveillance of statins-a descriptive analysis of psychiatric adverse reactions in eudravigilance. Pharm (Basel Switzerland). (2022) 15:1536. doi: 10.3390/ph15121536, PMID: 36558987 PMC9787673

[16] Shrivastava S Pucadyil TJ Paila YD Ganguly S Chattopadhyay A . Chronic cholesterol depletion using statin impairs the function and dynamics of human serotonin(1a) receptors. Biochemistry. (2010) 49:5426–35. doi: 10.1021/bi100276b, PMID: 20521763

[17] Repova K Aziriova S Krajcirovicova K Simko F . Cardiovascular therapeutics: A new potential for anxiety treatment? Medicinal Res Rev. (2022) 42:1202–45. doi: 10.1002/med.21875, PMID: 34993995 PMC9304130

[18] Zhang L Bao Y Tao S Zhao Y Liu M . The association between cardiovascular drugs and depression/anxiety in patients with cardiovascular disease: A meta-analysis. Pharmacol Res. (2022) 175:106024. doi: 10.1016/j.phrs.2021.106024, PMID: 34890773

[19] Molero Y Cipriani A Larsson H Lichtenstein P D’Onofrio BM Fazel S . Associations between statin use and suicidality, depression, anxiety, and seizures: A swedish total-population cohort study. Lancet Psychiatry. (2020) 7:982–90. doi: 10.1016/s2215-0366(20)30311-4, PMID: 33069320 PMC7606915

[20] Yang Q Yang Z Zeng B Jia J Sun F . Association of statin use with risk of depression and anxiety: A prospective large cohort study. Gen Hosp Psychiatry. (2024) 90:108–15. doi: 10.1016/j.genhosppsych.2024.07.015, PMID: 39106577

[21] Ye X Blais JE Ng VWS Castle D Hayes JF Wei Y . Association between statins and the risk of suicide attempt, depression, anxiety, and seizure: A population-based, self-controlled case series study. J Affect Disord. (2023) 320:421–7. doi: 10.1016/j.jad.2022.09.148, PMID: 36206879

[22] Chen Z Jiang T Peng Y Qiang X Yang F Hu H . Acupuncture and moxibustion treating lower urinary tract symptoms due to benign prostatic hyperplasia: A systematic review and network meta-analysis. Acupuncture Herbal Med. (2022) 2:84–90. doi: 10.1097/HM9.0000000000000029

[23] Page MJ McKenzie JE Bossuyt PM Boutron I Hoffmann TC Mulrow CD . The prisma 2020 statement: an updated guideline for reporting systematic reviews. BMJ (Clinical Res ed). (2021) 372:n71. doi: 10.1136/bmj.n71, PMID: 33782057 PMC8005924

[24] Zhu Z Yuan X Zheng Y Dou B Liu L Loh PY . Effectiveness of acupuncture in managing aromatase inhibitor-related arthralgia in breast cancer: A systematic review and meta-analysis. Acupuncture Herbal Med. (2025) 5:352–65. doi: 10.1097/HM9.0000000000000172

[25] Yeh JJ Syue SH Lin CL Hsu CY Shae Z Kao CH . Effects of statins on anxiety and depression in patients with asthma-chronic obstructive pulmonary disease overlap syndrome. J Affect Disord. (2019) 253:277–84. doi: 10.1016/j.jad.2019.05.002, PMID: 31071545

[26] Young-Xu Y Chan KA Liao JK Ravid S Blatt CM . Long-term statin use and psychological well-being. J Am Coll Cardiol. (2003) 42:690–7. doi: 10.1016/s0735-1097(03)00785-x, PMID: 12932603 PMC2673913

[27] Salagre E Fernandes BS Dodd S Brownstein DJ Berk M . Statins for the treatment of depression: A meta-analysis of randomized, double-blind, placebo-controlled trials. J Affect Disord. (2016) 200:235–42. doi: 10.1016/j.jad.2016.04.047, PMID: 27148902

[28] Kwak B Mulhaupt F Veillard N Pelli G Mach F . The hmg-coa reductase inhibitor simvastatin inhibits ifn-gamma induced mhc class ii expression in human vascular endothelial cells. Swiss Med weekly. (2001) 131:41–6. doi: 10.4414/smw.2001.06144, PMID: 11219190

[29] O’Neil A Sanna L Redlich C Sanderson K Jacka F Williams LJ . The impact of statins on psychological wellbeing: A systematic review and meta-analysis. BMC Med. (2012) 10:154. doi: 10.1186/1741-7015-10-154, PMID: 23206308 PMC3568015

[30] Vc J Balaji O Chogtu B . Effect of different statins in animal model of anxiety in wistar rats. Asian J Pharm Clin Res. (2018) 11:369–71. doi: 10.22159/ajpcr.2018.v11i10.24222

[31] Santos T Baungratz MM Haskel SP de Lima DD da Cruz JN Magro DD . Behavioral interactions of simvastatin and fluoxetine in tests of anxiety and depression. Neuropsychiatr Dis Treat. (2012) 8:413–22. doi: 10.2147/ndt.s31714, PMID: 23055736 PMC3464062

[32] Guo B Zhang M Hao W Wang Y Zhang T Liu C . Neuroinflammation mechanisms of neuromodulation therapies for anxiety and depression. Trans Psychiatry. (2023) 13:5. doi: 10.1038/s41398-022-02297-y, PMID: 36624089 PMC9829236

[33] Avan R Sahebnasagh A Hashemi J Monajati M Faramarzi F Henney NC . Update on statin treatment in patients with neuropsychiatric disorders. Life (Basel Switzerland). (2021) 11:1365. doi: 10.3390/life11121365, PMID: 34947895 PMC8703562

[34] Vevera J Valeš K Fišar Z Hroudová J Singh N Stuchlík A . The effect of prolonged simvastatin application on serotonin uptake, membrane microviscosity and behavioral changes in the animal model. Physiol Behav. (2016) 158:112–20. doi: 10.1016/j.physbeh.2016.02.029, PMID: 26917054

[35] Olmastroni E Molari G De Beni N Colpani O Galimberti F Gazzotti M . Statin use and risk of dementia or alzheimer’s disease: A systematic review and meta-analysis of observational studies. Eur J Prev Cardiol. (2022) 29:804–14. doi: 10.1093/eurjpc/zwab208, PMID: 34871380

[36] Yeni Y Cicek B Hacimuftuoglu A Ozkaraca M Lacin BB . Protective effect of hmg-coa reductase inhibitor rosuvastatin on doxorubicin-induced cognitive impairment, oxidative stress and neuroinflammation: possible role of creb, erk1/2, and BDNF. J neuroimmune pharmacology: Off J Soc NeuroImmune Pharmacol. (2025) 20:53. doi: 10.1007/s11481-025-10213-6, PMID: 40358798 PMC12075306

