# Supplementary material for: The association of statin use with the risk of anxiety: a systematic review and meta-analysis
Source: Front Psychiatry. 2026 Feb 18;17:1769044. doi: 10.3389/fpsyt.2026.1769044 (PMC12957783; doi:10.3389/fpsyt.2026.1769044)
Supplement: Supplementary file 1 [file DataSheet1.docx]

| **Supplementary Material Table S1** | |
| --- | --- |
| Number | Search Term |
| #1 | Hydroxymethylglutaryl-CoA Reductase Inhibitors[MeSH terms] OR Hydroxymethylglutaryl-CoA Reductase Inhibitors[Title/Abstract] OR Inhibitors, Hydroxymethylglutaryl-CoA Reductase[Title/Abstract] OR Reductase Inhibitors, Hydroxymethylglutaryl-CoA[Title/Abstract] OR HMG-CoA Reductase Inhibitor[Title/Abstract] OR HMG CoA Reductase Inhibitor[Title/Abstract] OR Statin[Title/Abstract] OR Statins[Title/Abstract] OR Inhibitors, HMG-CoA Reductase[Title/Abstract] OR Inhibitors, HMG CoA Reductase[Title/Abstract] OR Reductase Inhibitors, HMG-CoA[Title/Abstract] OR HMG-CoA Reductase Inhibitors[Title/Abstract] OR HMG CoA Reductase Inhibitors[Title/Abstract] OR Inhibitors, Hydroxymethylglutaryl-Coenzyme A[Title/Abstract] OR Hydroxymethylglutaryl-Coenzyme A Inhibitors[Title/Abstract] OR Inhibitors, Hydroxymethylglutaryl Coenzyme A[Title/Abstract] OR Inhibitors, Hydroxymethylglutaryl-CoA[Title/Abstract] OR Hydroxymethylglutaryl-CoA Inhibitors[Title/Abstract] OR Inhibitors, Hydroxymethylglutaryl CoA[Title/Abstract] OR Hydroxymethylglutaryl-CoA Reductase Inhibitor[Title/Abstract] OR Hydroxymethylglutaryl CoA Reductase Inhibitor[Title/Abstract] OR Reductase Inhibitor, Hydroxymethylglutaryl-CoA[Title/Abstract] OR Statins, HMG-CoA[Title/Abstract] OR HMG-CoA Statins[Title/Abstract] OR Statins, HMG CoA[Title/Abstract] OR Atorvastatin[Title/Abstract] OR Simvastatin[Title/Abstract] OR Rosuvastatin[Title/Abstract] OR Pravastatin[Title/Abstract] OR Pivalatedstatin[Title/Abstract] OR Fluvastatin[Title/Abstract] OR Lovastatin[Title/Abstract] |
| #2 | Anxiety[MeSH terms] OR Anxiety Disorders[MeSH terms] OR Anxiety[Title/Abstract] OR Anxiety Disorders[Title/Abstract] OR Angst[Title/Abstract] OR Nervousness[Title/Abstract] OR Hypervigilance[Title/Abstract] OR Social Anxiety[Title/Abstract] OR Anxieties, Social[Title/Abstract] OR Anxiety, Social[Title/Abstract] OR Social Anxieties[Title/Abstract] OR Anxiousness[Title/Abstract] OR Anxiety Disorder[Title/Abstract] OR Disorder, Anxiety[Title/Abstract] OR Disorders, Anxiety[Title/Abstract] OR Neuroses, Anxiety[Title/Abstract] OR Anxiety Neuroses[Title/Abstract] OR Anxiety States, Neurotic[Title/Abstract] OR Anxiety State, Neurotic[Title/Abstract] OR Neurotic Anxiety State[Title/Abstract] OR Neurotic Anxiety States[Title/Abstract] OR State, Neurotic Anxiety[Title/Abstract] OR States, Neurotic Anxiety[Title/Abstract] |
| #3 | risk[Title/Abstract] OR incidence[Title/Abstract] OR association[Title/Abstract] OR cohort[Title/Abstract] OR case-control[Title/Abstract] OR “odds ratio”[Title/Abstract] OR “hazard ratio”[Title/Abstract] OR “relative risk”[Title/Abstract] |
| #4 | #1 AND #2 AND #3 |
